# A machine learning framework for multimodal temporal prediction of neurological outcome after out-of-hospital cardiac arrest

**DOI:** 10.1038/s41598-026-59052-2

**Published:** 2026-06-23

**Authors:** Jee Yong Lim, Han Joon Kim

**Affiliations:** 1 International Healthcare Center, Seoul St. Mary’s Hospital, College of Medicine, The Catholic University of Korea, Seoul, Republic of Korea; 2 Department of Emergency Medicine, Seoul St. Mary’s Hospital, College of Medicine, The Catholic University of Korea, 222 Banpo-daero, Seocho-gu, Seoul, 06591 Republic of Korea

**Keywords:** Cardiac arrest, Neurological prognostication, Machine learning, Multimodal prediction, Thromboinflammation, Explainable artificial intelligence, Targeted temperature management, Biomarkers, Medical research, Neurology, Neuroscience

## Abstract

Neurological prognostication after out-of-hospital cardiac arrest (OHCA) remains challenging. Existing clinical scores rely on static, single-timepoint assessments and fail to capture the dynamic interplay among coagulation derangement, systemic inflammation, brain injury, and evolving neurological status. Whether integrating serial multimodal data through machine learning can meaningfully improve prediction over established approaches has not been systematically evaluated. We conducted a retrospective cohort study of 414 consecutive OHCA patients treated with targeted temperature management (TTM) at a tertiary cardiac arrest center in South Korea (2009–2021), where withdrawal of life-sustaining treatment is not practiced. Ninety-one features spanning five modalities—coagulation, inflammation, brain injury biomarkers, neurological examination, and static clinical variables—were extracted at admission, 24 h, and 48 h. We compared four machine learning algorithms against single-modality models and a clinical score approximation using five-fold stratified cross-validation. Dynamic prediction models evaluated discriminative performance evolution. SHapley Additive exPlanations (SHAP) analysis quantified feature- and modality-level contributions. Robustness was assessed through temporal validation, self-fulfilling prophecy sensitivity analyses, and exclusion of clinician-decision-dependent variables. Of 414 patients (mean age 55.6 years, 71.8% male), 131 (31.6%) achieved favorable neurological outcome (Cerebral Performance Category [CPC] 1–2) at six months. The full multimodal random forest model achieved an area under the receiver operating characteristic curve (AUROC) of 0.983 (95% CI 0.972–0.991), significantly outperforming the clinical score approximation (AUROC 0.847; ΔAUROC + 0.136, *p* < 0.001) and every single-modality model (all *p* < 0.001). At 100% specificity, sensitivity was 0.519. Dynamic prediction improved from AUROC 0.950 at admission to 0.977 at 24 h (*p* < 0.001) and 0.981 at 48 h. SHAP analysis revealed that neurological examination and brain injury biomarkers dominated overall prediction, while coagulation markers—particularly initial international normalized ratio (INR)—provided the strongest early discriminative signal. The model remained robust on temporal validation (AUROC 0.977), after neurological examination exclusion (0.965), and after excluding clinician-decision-dependent variables (0.979). A multimodal machine learning framework integrating serial thromboinflammation, brain injury, and neurological data substantially outperforms conventional approaches for neurological prognostication after OHCA. The dynamic prediction capability and modality-level explainability offer a pathway toward clinically actionable, time-evolving decision support in post-cardiac arrest care.

**Supplementary Information:**

The online version contains supplementary material available at https://doi.org/10.1038/s41598-026-59052-2.

## Introduction

Out-of-hospital cardiac arrest (OHCA) is a leading cause of death and acquired disability worldwide, affecting over 350,000 individuals annually in Europe and a comparable number in North America^1,2^. Despite advances in resuscitation science, fewer than 10% of OHCA patients survive to hospital discharge with favorable neurological function. For those who achieve return of spontaneous circulation (ROSC) and are admitted to intensive care, the clinical trajectory is dominated by post-cardiac arrest syndrome (PCAS)—a complex pathophysiological state encompassing systemic ischemia-reperfusion injury, myocardial dysfunction, and hypoxic-ischemic brain injury^3^.

Accurate neurological prognostication is central to the management of comatose PCAS patients, directly informing decisions about continuation or withdrawal of life-sustaining treatment (WLST), resource allocation, and family counseling^4^. Premature prognostication has been identified as a contributor to self-fulfilling prophecy, in which the act of prognostication itself influences outcome by triggering treatment limitation^5^.

Current European Resuscitation Council (ERC) and American Heart Association (AHA) guidelines recommend a multimodal prognostication strategy combining clinical neurological examination, electrophysiology, somatosensory evoked potentials (SSEP), neuroimaging, and serum biomarkers, evaluated no earlier than 72 h after ROSC^4,6^. Several clinical prediction scores have been developed to standardize this assessment. The OHCA score, incorporating initial cardiac rhythm, no-flow time, and serum lactate, has been validated with AUROCs of 0.82–0.85^7^. The Cardiac Arrest Hospital Prognosis (CAHP) score, using arrest circumstances and initial pH, achieves AUROCs of 0.87–0.93^8^. While useful, these instruments share fundamental limitations: reliance on variables at a single timepoint, independent treatment of each modality, and static risk estimates that do not evolve as clinical information accumulates.

The pathophysiology of PCAS is a dynamic, multi-system process. Whole-body ischemia-reperfusion triggers coagulation cascade activation through tissue factor expression and endothelial injury, leading to a spectrum of coagulopathy from mild clotting time prolongation to disseminated intravascular coagulation^9,10^. Simultaneously, a systemic inflammatory response is initiated, characterized by leukocyte activation and endothelial dysfunction^3,11^. These thromboinflammatory processes interact bidirectionally—thrombin activates complement and inflammatory pathways, while inflammatory cytokines promote tissue factor expression—creating a self-amplifying cycle that may exacerbate secondary brain injury^12^. Serial measurements of coagulation markers (international normalized ratio [INR], antithrombin III, D-dimer) and inflammatory indices (neutrophil-to-lymphocyte ratio [NLR], C-reactive protein [CRP]) have been individually associated with neurological outcome^13,14^, yet these temporal patterns have not been integrated with established prognostic modalities within a unified framework.

Machine learning methods can model complex, nonlinear interactions among high-dimensional temporal data and have shown promise in critical care prognostication^15,16^. In cardiac arrest, machine learning has been applied using various combinations of clinical variables, electroencephalography (EEG), and biomarkers^17,18^. However, prior applications have largely focused on single-modality inputs, lacked temporal integration, or offered insufficient explainability for clinical adoption. SHapley Additive exPlanations (SHAP) provides a rigorous framework for attributing predictions to individual features and entire data modalities^19,20^.

In this study, we set out to test whether a multimodal machine learning framework—drawing on serial thromboinflammation markers, brain injury biomarkers (neuron-specific enolase [NSE], S100 calcium-binding protein [S100B], lactate), neurological examination (Glasgow Coma Scale [GCS], pupillary and corneal reflexes), and static clinical variables—could outperform conventional clinical scores and single-modality models for predicting six-month neurological outcome after OHCA. Beyond prediction accuracy, we examined three capabilities: dynamic prediction improving as serial data accumulate over the first 48 h, modality-level explainability through SHAP analysis, and robustness across multiple sensitivity analyses.

To our knowledge, this represents the first attempt to integrate serial thromboinflammation trajectories within a comprehensive, five-modality prediction system for post-cardiac arrest prognostication, with formal assessment of dynamic prediction capability and modality-level explainability.

## Methods

### Study design and population

This retrospective cohort study was conducted at Seoul St. Mary’s Hospital, a university-affiliated tertiary cardiac arrest center in Seoul, Republic of Korea. Consecutive adult (≥ 18 years) non-traumatic OHCA patients who achieved sustained ROSC and were treated with targeted temperature management (TTM) between January 2009 and December 2021 were included. Patients without six-month neurological outcome data were excluded (*n*= 5 of 419 screened). Data collection for this study was completed in 2022, with six-month follow-up for the last enrolled patients completed by mid-2022; subsequent data fell outside the approved study protocol. The study was conducted according to the Declaration of Helsinki and approved by the Institutional Review Board of the Catholic University of Korea, Seoul St. Mary’s Hospital (KC23RISI0264; approved 21 April 2023), with informed consent waived. The study was reported following the Transparent Reporting of a multivariable prediction model for Individual Prognosis Or Diagnosis (TRIPOD) guidelines (Type 1b)^21^.

### Clinical context: life-sustaining treatment

An important feature of this cohort is the clinical context regarding WLST. In South Korea, withdrawal of life-sustaining treatment in post-cardiac arrest patients is not practiced in routine clinical care. Unlike many Western intensive care unit (ICU) settings where prognostic assessment frequently leads to WLST—accounting for a substantial proportion of deaths—Korean medicolegal norms do not permit active treatment withdrawal in patients who have undergone resuscitation and intensive care. All patients in this cohort received full TTM and continued intensive care support throughout their hospitalization. Deaths occurred despite continued maximal treatment, reflecting the natural course of severe hypoxic-ischemic brain injury rather than treatment limitation decisions^5,22^.

### TTM protocol and sedation

TTM was initiated after ROSC using surface cooling (Arctic Sun, Medivance, Inc.) or endovascular cooling (Thermogard XP, Zoll Medical Corporation), targeting 33–36 °C per institutional protocol. The target temperature evolved over the study period: 33 °C predominated in earlier years (2009–2014), with 36 °C becoming more common after publication of the TTM and TTM2 trials^23^. The protocol consisted of induction, 24-hour maintenance, and controlled rewarming (0.25 °C/hour). Sedation during TTM employed midazolam for continuous sedation and rocuronium for neuromuscular blockade as first-line agents; fentanyl and propofol were used as adjuncts at physician discretion. All sedation and neuromuscular blockade were discontinued at the completion of rewarming; neurological assessments were performed after cessation of these agents.

### Data collection

Clinical data were extracted from a prospectively maintained cardiac arrest registry supplemented by electronic medical record review. Serial laboratory measurements were obtained at admission (T0), 12 h, 24 h, 48 h, and 72 h after ROSC. The 12-hour timepoint was excluded due to 77–94% missing data across coagulation markers, reflecting inconsistent sampling during active TTM induction. The 72-hour timepoint was excluded for markers with < 50% availability (fibrinogen 7%, antithrombin III [ATIII] 10%, D-dimer 11%, fibrin degradation products [FDP] 9%), yielding three primary timepoints (T0, T24, T48).

### Outcome definition

The primary outcome was six-month neurological status assessed using the Cerebral Performance Category (CPC) scale by treating physicians or structured telephone follow-up^24^. Favorable outcome: CPC 1–2 (good or moderate disability). Poor outcome: CPC 3–5 (severe disability, vegetative state, or death). Models were trained to predict a binary outcome (1 = favorable, 0 = poor).

### Feature engineering

Ninety-one features were engineered across five modalities at three serial timepoints, capturing raw values and within-patient trajectory changes (Δ features):

*Modality 1—Coagulation (28 features)* INR, prothrombin time (PT), activated partial thromboplastin time (aPTT), ATIII, D-dimer, FDP at T0/T24/T48 (18 raw) plus Δ24h and Δ48h trajectory features for five core markers (10 derived).

*Modality 2—Inflammation (19 features)* White blood cell count, neutrophil%, lymphocyte%, CRP at T0/T24/T48 (12 raw), NLR at each timepoint (3), and Δ48h trajectories (4).

*Modality 3—Brain injury biomarkers (14 features)* NSE and S100B at T0/T24/T48 (6 raw), Δ48h trajectories and peak values (4), initial and 24 h lactate with 24 h clearance (4).

*Modality 4—Neurological examination (11 features)* GCS total at T0/T24/T48, GCS Δ48h, pupillary light reflex (PLR) and corneal reflex (CR) at T0/T24/T48 (coded 0–2), spontaneous respiration.

*Modality 5—Static clinical variables (19 features)* Age, sex, 7 comorbidities, 5 arrest characteristics (including derived shockable rhythm), TTM target temperature, ST-elevation myocardial infarction (STEMI), coronary angiography, percutaneous coronary intervention (PCI), SSEP result (bilateral N20 presence/absence), and amplitude-integrated EEG (aEEG) monitoring availability. SSEP encodes the examination result; aEEG encodes availability rather than classification, as standardized pattern reporting was inconsistent across the 13-year study period.

### Missing data

Missing values were imputed with feature-wise medians. For binary categorical variables (sex, comorbidities, arrest characteristics), median imputation is equivalent to mode imputation and assigns the majority class value. While data were unlikely to be missing completely at random—laboratory sampling frequency may correlate with clinical severity—median imputation was chosen for its robustness with tree-based ensemble methods, which partition feature space through binary splits and are inherently less sensitive to imputed values than distance-based or linear models. We acknowledge that more sophisticated approaches such as multiple imputation by chained equations (MICE) could better preserve uncertainty structure, and this is discussed as a limitation. Features were excluded if an entire timepoint had > 75% missing (12 h timepoint) or if individual marker availability was < 50% at a given timepoint (72 h for ATIII, D-dimer, fibrinogen, FDP). Among the retained 91 features, median missingness was 12.1% (mean 15.9%). Modality-level data availability (≥ 50% threshold): coagulation 87.4%, inflammation 89.1%, brain injury 85.0%, neurological examination 98.3%, static 99.5%.

### Machine learning models

Four algorithms were compared: random forest (300 trees, max depth 6, class-weight balanced)^25^; extreme gradient boosting (XGBoost; 300 rounds, max depth 4, learning rate 0.05, L1/L2 regularization, scale_pos_weight 2.16)^26^; gradient boosting machine (GBM; 300 estimators, max depth 4); and L2-regularized logistic regression (C = 0.1, standardized inputs). Conservative hyperparameters were chosen to mitigate overfitting given the moderate sample size.

### Evaluation and benchmarks

All models were evaluated using five-fold stratified cross-validation. Out-of-fold predicted probabilities were aggregated across all five folds, yielding a single prediction per patient that was never used during training. To ensure no data leakage, the cross-validation procedure was implemented with strict separation: at each fold, the model was trained exclusively on training-fold patients and generated predictions only for held-out patients; imputation medians were computed within the training fold; and no feature was derived from the outcome variable. Performance metrics were computed once on this full set of pooled predictions. An OHCA-like clinical score benchmark was constructed using logistic regression with four baseline features (shockable rhythm, anoxic time, initial lactate, age) approximating key components of established scores; this represents a simplified proxy, as arterial pH and epinephrine dose were unavailable. Single-modality XGBoost models (200 estimators, max depth 3) quantified each modality’s independent contribution.

### Dynamic prediction

Three cumulative models were trained at clinically meaningful timepoints: T0 (admission; 37 features), T24 (0–24 h; 60 features), T48 (0–48 h; 91 features). All 414 patients were included at each timepoint; missing features were imputed with medians.

### Explainability analysis

SHAP values were computed using TreeExplainer on a full-data XGBoost model^20^. Feature importance: mean |SHAP|. Modality importance: sum of absolute SHAP across constituent features per patient, averaged across the cohort.

### Sensitivity analyses

Five pre-specified sensitivity analyses were performed: (1) neurological examination exclusion (80 features); (2) temporal validation (train 2009–2017, *n* = 304; test 2018–2021, *n* = 110); (3) early death (< 72 h) exclusion; (4) thromboinflammation-only model (Modalities 1 + 2; 47 features); and (5) clinician-decision-dependent variable exclusion (SSEP, aEEG, angiography, PCI removed; 87 features).

### Statistics and reproducibility

Primary metric: AUROC with bootstrap 95% CI (1,000 resamples). Secondary: area under the precision-recall curve (AUPRC) with CI, Brier score, and sensitivity at 100% and 95% specificity. Between-model comparisons: bootstrap DeLong-equivalent test (2,000 resamples). Baseline comparisons: Mann–Whitney U (continuous) and chi-square (categorical). All tests two-sided, α = 0.05. Analyses performed in Python 3.12 (scikit-learn v1.4, XGBoost v2.0, SHAP v0.44). Random seed 42^27^.

## Results

### Study population

**Fig. 1:**
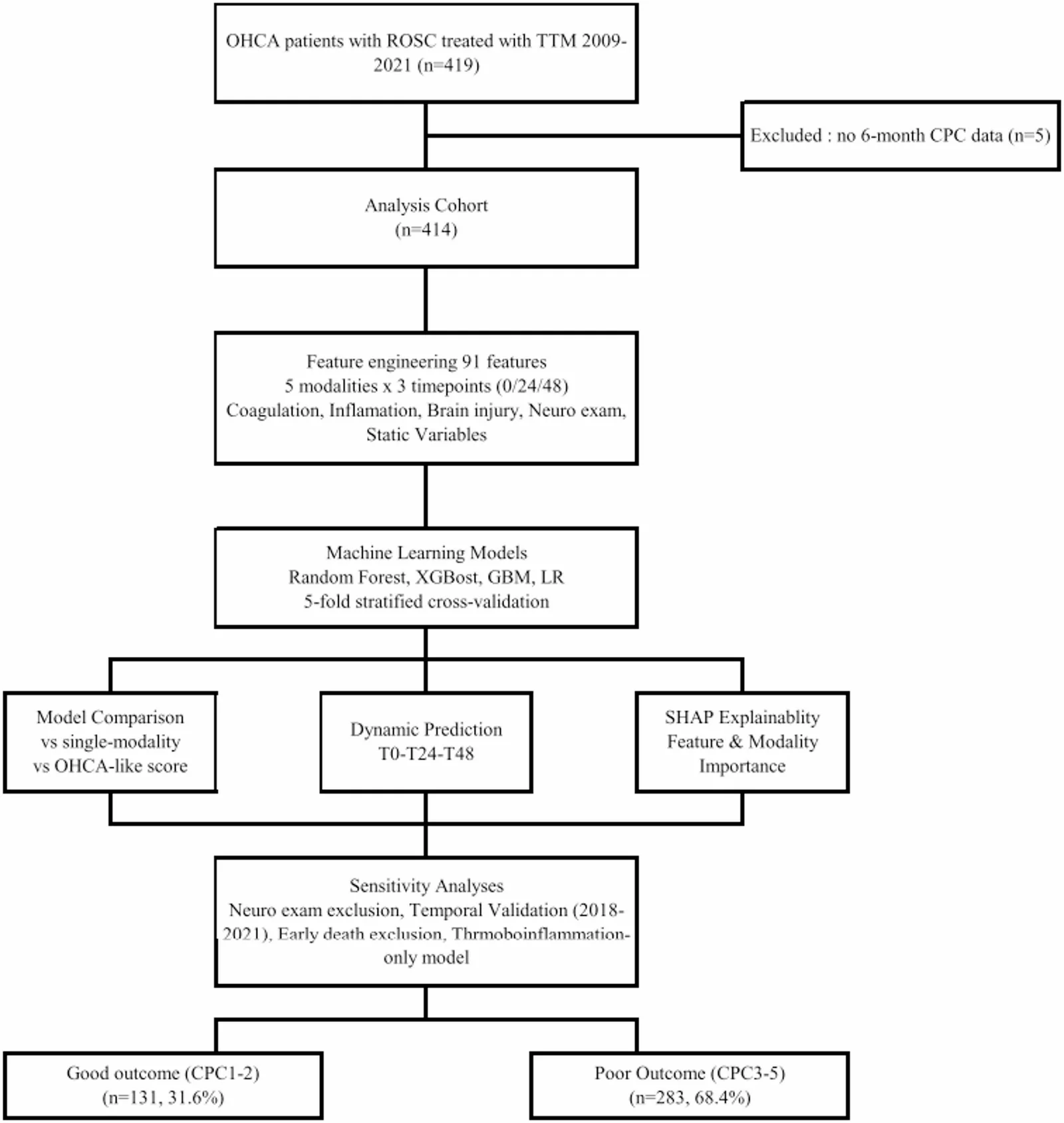
Study flow diagram showing patient selection and analytical workflow. OHCA, out-of-hospital cardiac arrest; ROSC, return of spontaneous circulation; TTM, targeted temperature management; CPC, Cerebral Performance Category.

Between January 2009 and December 2021, 419 consecutive adult OHCA patients treated with TTM were screened, of whom 5 were excluded for missing six-month follow-up data, yielding an analytical cohort of 414 patients (Fig. 1a). The mean age was 55.6 ± 16.4 years, and 301 (72.7%) were male. A shockable initial rhythm was present in 169 (40.8%), 291 (70.3%) had witnessed arrests, and 264 (63.8%) received bystander cardiopulmonary resuscitation (CPR). At six-month follow-up, 131 (31.6%) achieved favorable outcome (CPC 1–2) and 283 (68.4%) had poor outcome (CPC 3–5). Among patients with poor outcome, 182 died during hospitalization despite continued full intensive care support, and 101 survived with severe disability (CPC 3–4). Of the 182 deaths, 49 (26.9%) occurred within 72 h. Do-not-attempt-resuscitation orders were present in 5 patients (1.2%) at admission. Baseline characteristics are presented in Table 1.

**Table 1:** Baseline characteristics stratified by six-month neurological outcome.

Characteristic	Total (*n* = 414)	Good (*n* = 131)	Poor (*n* = 283)	*p*
Age, years (mean ± SD)	55.6 ± 16.4	50.3 ± 14.5	58.1 ± 16.8	< 0.001
Male sex, n (%)	301 (72.7)	106 (80.9)	195 (68.9)	0.010
Shockable rhythm, n (%)	169 (40.8)	94 (71.8)	75 (26.5)	< 0.001
Witnessed arrest, n (%)	291 (70.3)	101 (77.1)	190 (67.1)	0.040
Bystander CPR, n (%)	264 (63.8)	95 (72.5)	169 (59.7)	0.013
Cardiac etiology, n (%)	208 (50.2)	92 (70.2)	116 (41.0)	< 0.001
Hypertension, n (%)	165 (39.9)	43 (32.8)	122 (43.1)	0.048
Diabetes mellitus, n (%)	112 (27.1)	25 (19.1)	87 (30.7)	0.014
GCS at admission, median [IQR]	3 [3–3]	3 [3–4]	3 [3–3]	0.019
INR at admission	1.2 [1.1–1.4]	1.1 [1.0–1.2]	1.3 [1.1–1.5]	< 0.001
NSE at admission, µg/L	32.4 [18.6–58.7]	24.2 [15.3–36.0]	39.7 [21.5–71.4]	< 0.001
Lactate at admission, mmol/L	8.5 [4.3–12.7]	6.2 [3.1–9.8]	9.8 [5.2–13.6]	< 0.001
Survival to discharge, n (%)	260 (62.8)	131 (100)	129 (45.6)	< 0.001

Values are mean ± SD, median [IQR], or n (%). Good: CPC 1–2; Poor: CPC 3–5. P values: Mann–Whitney U test (continuous), chi-square test (categorical).

### Data completeness

Across 91 features, median data availability was 87.9%. Data availability per dynamic prediction timepoint: T0 median 97.3% (all 414 patients with ≥ 50% features available), T24 median 95.0% (410/414 with ≥ 50%), T48 median 91.2% (374/414 with ≥ 50%). All 414 patients were included in all models; missing values were imputed with medians.

### Multimodal model performance

The full multimodal random forest achieved the highest AUROC of 0.983 (95% CI 0.972–0.991), with AUPRC 0.964 (0.942–0.982) and Brier score 0.065 (Table 2). XGBoost was comparable in discrimination (AUROC 0.981 [0.968–0.991]) with better calibration (Brier 0.050, calibration slope 0.96). GBM achieved AUROC 0.976 (0.961–0.987), and logistic regression 0.972 (0.958–0.984). At 100% specificity, the random forest achieved sensitivity of 0.519 and logistic regression 0.550, meaning that over half of favorable-outcome patients were correctly identified with zero false positives for poor outcome prediction. At 95% specificity, random forest sensitivity was 0.924. The OHCA-like benchmark achieved AUROC 0.847 (0.806–0.883) and sensitivity of 0.061 at 100% specificity.

**Table 2:** Predictive performance of multimodal, single-modality, and benchmark models.

Model	AUROC	95% CI	AUPRC	95% CI	Brier	S@100	ΔAUROC
Full models							
RF	0.983	0.972–0.991	0.964	0.942–0.982	0.065	0.519	Ref
XGBoost	0.981	0.968–0.991	0.955	0.921–0.980	0.050	0.244	−0.002
GBM	0.976	0.961–0.987	0.947	0.914–0.974	0.058	0.275	−0.007
LR	0.972	0.958–0.984	0.945	0.917–0.969	0.063	0.550	−0.011*
Single modality							
Neuro exam	0.946	0.920–0.969	0.894	0.836–0.940	—	—	−0.037***
Brain injury	0.936	0.912–0.958	0.838	0.768–0.902	—	—	−0.047***
Static	0.860	0.819–0.897	0.698	0.621–0.796	—	—	−0.123***
Coagulation	0.832	0.791–0.871	0.708	0.629–0.786	—	—	−0.151***
Inflammation	0.703	0.655–0.753	0.494	0.422–0.590	—	—	−0.280***
Benchmark							
OHCA-like	0.847	0.806–0.883	0.718	0.639–0.794	—	0.061	−0.136***
Dynamic							
T0 (0 h)	0.950	0.931–0.968	0.886	0.832–0.931	—	0.122	—
T24 (0–24 h)	0.977	0.965–0.987	0.948	0.912–0.974	—	0.252	—
T48 (0–48 h)	0.981	0.970–0.991	0.955	0.926–0.981	—	0.244	—

AUROC, area under the receiver operating characteristic curve; AUPRC, area under the precision-recall curve; S@100, sensitivity at 100% specificity; ΔAUROC, difference from full random forest. CIs from 1,000 bootstrap resamples. *p < 0.05, ***p < 0.001 (bootstrap DeLong-equivalent test, 2,000 resamples). Note: overlapping CIs do not preclude significant paired differences, as the DeLong test evaluates the paired difference distribution.

The multimodal model significantly outperformed every single-modality model (Fig. 3a–b): neurological examination (AUROC 0.946 [0.920–0.969]; ΔAUROC − 0.037, *p* < 0.001), brain injury biomarkers (0.936 [0.912–0.958]; −0.047, *p* < 0.001), static variables (0.860 [0.819–0.897]; −0.123, *p* < 0.001), coagulation (0.832 [0.791–0.871]; −0.151, *p* < 0.001), and inflammation (0.703 [0.655–0.753]; −0.280, *p* < 0.001).

### Model calibration

**Fig. 2:**
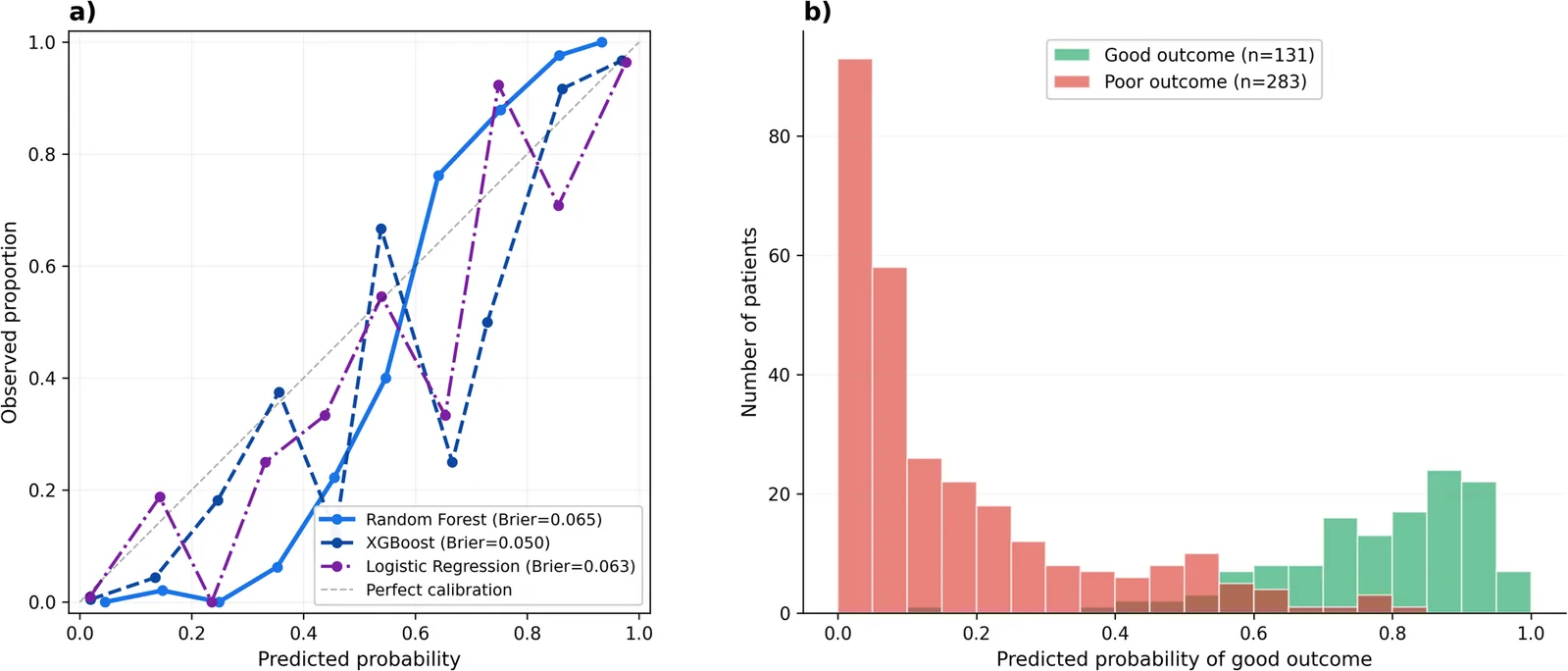
Model calibration assessment. (**a**) Calibration curves (reliability diagrams) for the three primary models; dashed line indicates perfect calibration; Brier scores shown in legend. (**b**) Distribution of predicted probabilities from the random forest model, stratified by observed outcome (green bars, favorable outcome; red bars, poor outcome).

XGBoost demonstrated the best calibration (Brier 0.050; calibration slope 0.96, intercept − 0.08), with predicted probabilities closely tracking observed event rates across risk deciles (Fig. 2a). Logistic regression was also well calibrated (Brier 0.063; slope 1.00). The random forest showed slight miscalibration at intermediate probabilities (Brier 0.065; slope 1.36). The distribution of predicted probabilities (Fig. 2b) showed good separation between outcome groups, with most poor-outcome patients receiving probabilities below 0.2 and most favorable-outcome patients above 0.7. A clinically relevant overlap zone existed between 0.2 and 0.5.

### Dynamic prediction

**Fig. 3:**
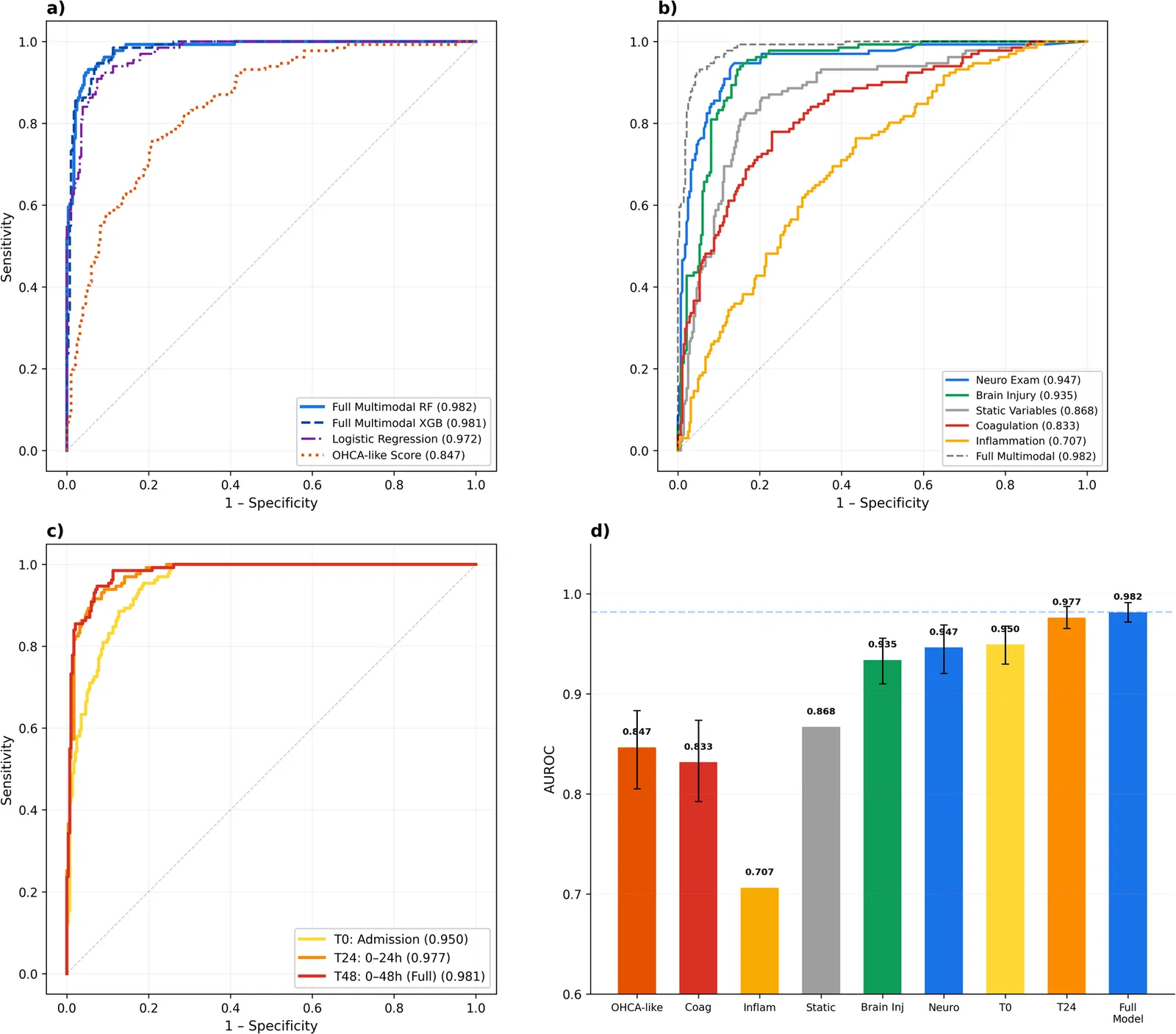
Model performance comparison. (**a**) Receiver operating characteristic (ROC) curves for full multimodal models (random forest in solid blue, XGBoost in dashed dark blue, logistic regression in dash-dot purple) and the OHCA-like score benchmark (dotted orange). (**b**) Single-modality ROC curves versus full multimodal model (dashed black line). (**c**) Dynamic prediction ROC curves at T0 (admission, yellow), T24 (24 h, orange), and T48 (48 h, red). (**d**) AUROC comparison with 95% bootstrap confidence intervals.

Dynamic prediction showed progressive improvement as data accumulated (Fig. 3c–d). At admission (T0, 37 features), AUROC was 0.950 (0.931–0.968). Adding 24-hour data (T24, 60 features) improved performance to 0.977 (0.965–0.987; ΔAUROC + 0.027, *p* < 0.001 vs. T0). The full 48-hour model (T48, 91 features) reached 0.981 (0.970–0.991; ΔAUROC + 0.004 vs. T24, *p* = 0.252). The T0–to–T24 improvement was statistically significant, while the T24–to–T48 gain was not.

### Explainability analysis

**Fig. 4:**
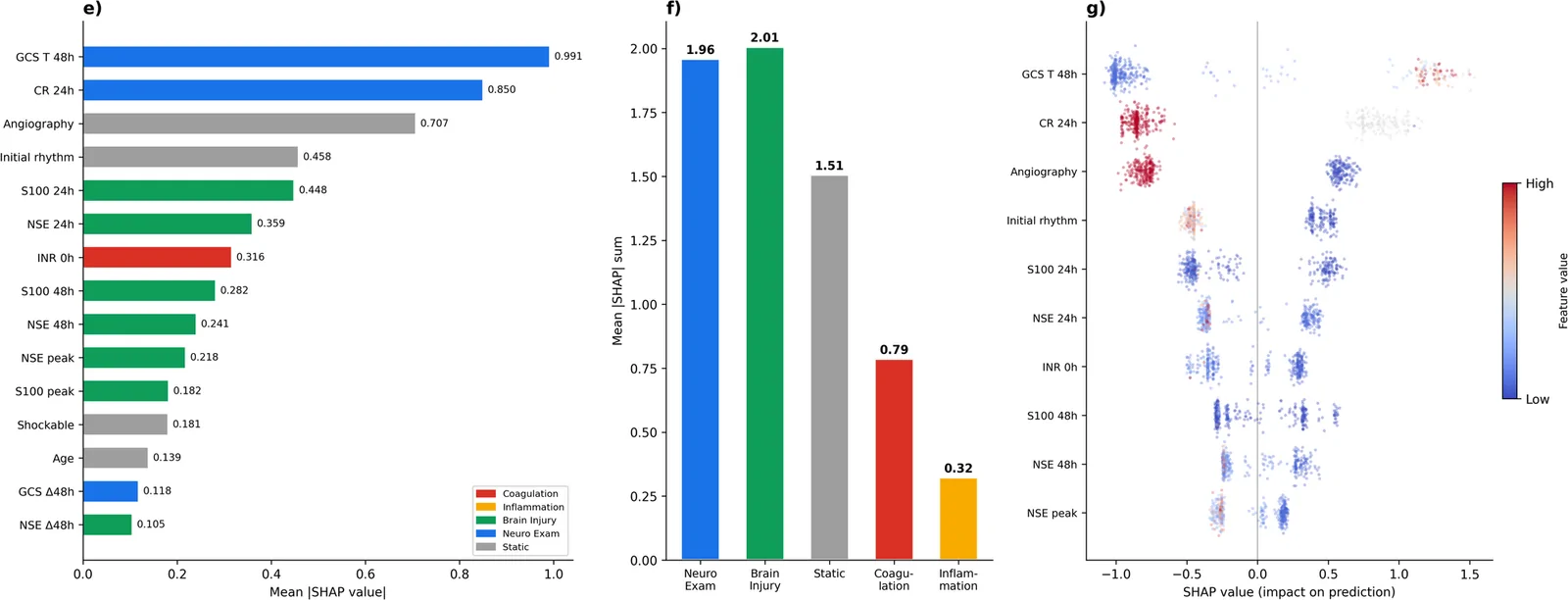
Explainability analysis. (**a**) Feature-level SHAP importance for top 15 predictors, colored by modality: coagulation (red), inflammation (yellow), brain injury (green), neurological examination (blue), static (gray). (**b**) Modality-level aggregate SHAP importance. (**c**) SHAP beeswarm plot for top 10 features; color indicates feature value (blue, low; red, high); horizontal position indicates direction and magnitude of effect. SHAP, SHapley Additive exPlanations; GCS, Glasgow Coma Scale; CR, corneal reflex; INR, international normalized ratio; NSE, neuron-specific enolase; PLR, pupillary light reflex; ATIII, antithrombin III; PT, prothrombin time.

SHAP analysis revealed a hierarchy of modality importance (Fig. 4a–b). Brain injury biomarkers contributed the highest aggregate importance (mean |SHAP| sum = 2.006), followed by neurological examination (1.959), static clinical variables (1.506), coagulation (0.786), and inflammation (0.323). At the individual feature level, the top five predictors were 48-hour GCS total (mean |SHAP| = 0.991), 24-hour corneal reflex (0.850), coronary angiography (0.707), initial cardiac rhythm (0.458), and 24-hour S100B (0.448). Among coagulation markers, initial INR ranked 7th overall (|SHAP| = 0.316). The beeswarm plot (Fig. 4c) showed that higher INR, elevated NSE and S100B, and lower GCS pushed predictions toward poor outcome, while shockable rhythm, intact pupillary reflexes, and higher ATIII favored good outcome predictions.

### Sensitivity analyses

**Fig. 5:**
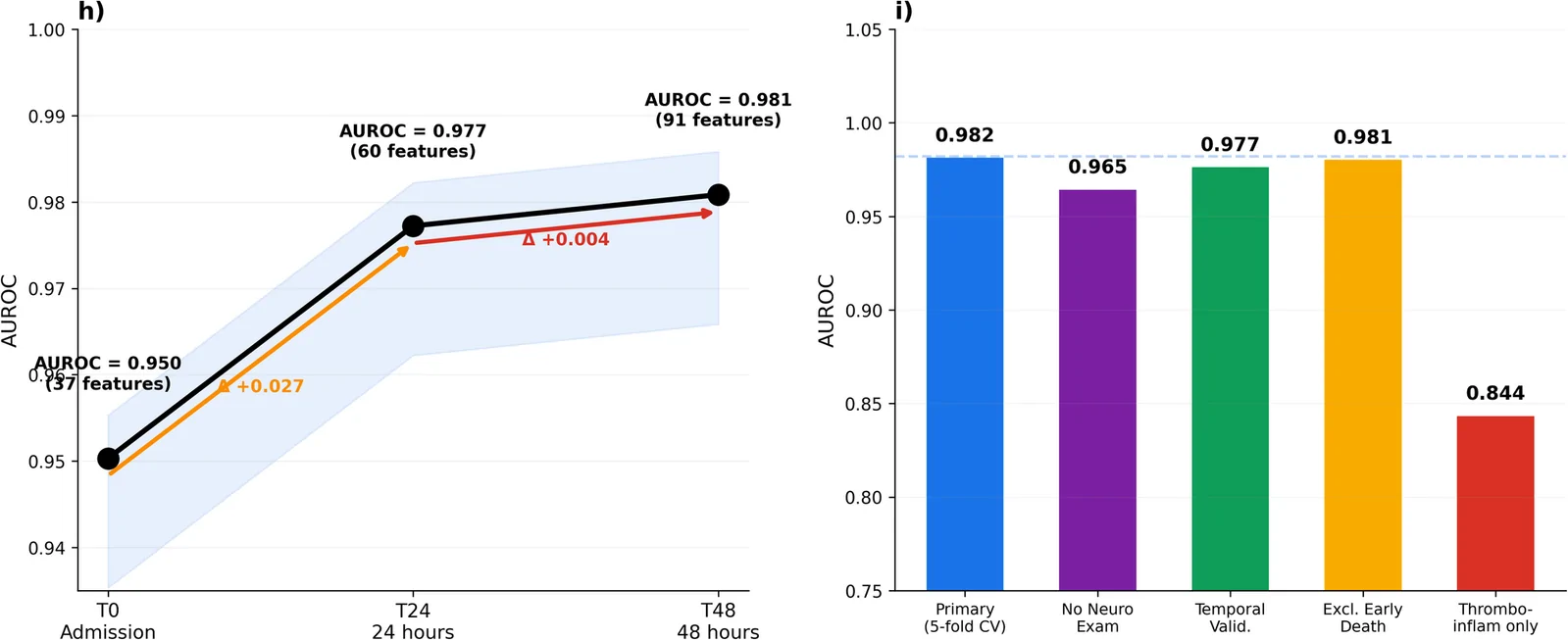
Dynamic prediction and sensitivity analyses. (**a**) AUROC trajectory from admission through 48 h, with incremental changes (ΔAUROC) annotated. (**b**) Sensitivity analysis results showing AUROC for the primary model, neurological examination exclusion, temporal validation (2018–2021 test set), early death exclusion, and thromboinflammation-only model.

The model demonstrated consistent performance across all five sensitivity analyses (Table 3; Fig. 5). Excluding neurological examination features yielded AUROC 0.965 (80 features). Excluding clinician-decision-dependent variables (SSEP, aEEG, angiography, PCI) yielded 0.979 (87 features). Temporal validation (train 2009–2017, *n* = 304; test 2018–2021, *n* = 110; 30.9% favorable outcome) yielded 0.977. Excluding 49 patients with early death before 72 h yielded 0.981 (*n* = 365). The thromboinflammation-only model achieved 0.844 (47 features).

**Table 3:** Sensitivity analyses.

Sensitivity analysis	*N*	AUROC	Features	Good (%)
Primary (5-fold CV)	414	0.983	91	31.6
Excl neuro exam (M4)	414	0.965	80	31.6
Excl clinician-decision vars	414	0.979	87	31.6
Temporal valid (2018–2021)	110	0.977	91	30.9
Excl early death (< 72 h)	365	0.981	91	35.9
Thromboinflam only (M1 + M2)	414	0.844	47	31.6

M1, coagulation; M2, inflammation; M4, neurological examination. Clinician-decision variables: SSEP, aEEG, angiography, PCI. T0–T24 ΔAUROC + 0.027, *p* < 0.001; T24–T48 ΔAUROC + 0.004, *p* = 0.252.

## Discussion

In this study, we developed and internally validated a multimodal machine learning framework for neurological prognostication after OHCA that integrates serial thromboinflammation markers, brain injury biomarkers, neurological examination, and static clinical variables across the first 48 h of post-resuscitation care. Several findings merit discussion. The multimodal approach achieved an AUROC of 0.983, a clear improvement over both conventional scoring approaches and any single data modality used in isolation. Dynamic prediction showed that discrimination improves as data accumulate, with the steepest gains in the first 24 h. SHAP analysis uncovered a temporal hierarchy of modality importance—coagulation markers contributed most as early discriminators, before brain injury biomarker trajectories had time to diverge. In addition, the model held up well across all pre-specified sensitivity analyses, including temporal validation, exclusion of neurological examination features, and exclusion of clinician-decision-dependent variables.

The AUROC of 0.983 compares favorably with previously reported models. The OHCA score has been validated with AUROCs of 0.82–0.85, the CAHP score at 0.87–0.93, and the TTM score at approximately 0.84^7,8^. Machine learning approaches using clinical variables alone have typically achieved AUROCs of 0.85–0.92^17,18^. The magnitude of improvement over the clinical score benchmark (ΔAUROC + 0.136) suggests that integrating serial multimodal data captures information that single-timepoint scoring systems miss. The near-equivalent performance of logistic regression (0.972) and tree-based methods (0.981–0.983) indicates that much of the prognostic signal is capturable through relatively straightforward modeling. At 100% specificity, the random forest correctly identified 51.9% of favorable-outcome patients with zero false positives, a clinically meaningful threshold for ruling in good prognosis^28^.

A distinguishing feature of our study is the clinical context regarding self-fulfilling prophecy. In contrast to Western ICU settings where WLST based on early prognostic assessment is a recognized pathway to death, withdrawal of life-sustaining treatment is not practiced for post-cardiac arrest patients in South Korea. All 182 deaths in our cohort occurred despite continued full intensive care support, and do-not-attempt-resuscitation orders were present in only 5 patients (1.2%) at admission. This means that the self-fulfilling prophecy—in which prognostic assessment triggers WLST, which causes the predicted poor outcome—does not operate in our cohort^5,22^. We believe this represents a meaningful methodological strength compared to studies from settings where WLST is prevalent. Additionally, excluding all neurological examination features—the modality most susceptible to clinician bias—resulted in an AUROC of only 0.965, a decline of just 0.018, demonstrating that objective laboratory-based data alone carry most of the prognostic signal. Excluding 49 patients with early death (< 72 h) had no effect (AUROC 0.981), and temporal validation showed stable performance across the 2018–2021 test era despite evolving TTM protocols.

From a clinical standpoint, the dynamic prediction results may be the most practically relevant aspect of this work. Current guidelines advise against prognostication before 72 h, yet the T0 model achieved an AUROC of 0.950 at admission using only arrest characteristics, baseline laboratory values, and static variables. The improvement to 0.977 at 24 h (ΔAUROC + 0.027, *p* < 0.001) was driven by the first serial NSE, S100B, and coagulation trajectory data becoming available. After 24 h, the additional gain was not statistically significant (ΔAUROC + 0.004, *p*= 0.252), consistent with evidence that biomarker trajectories during the first day capture most of the relevant information about secondary brain injury^29^. This temporal framework aligns with the emerging paradigm of real-time, continuous physiological monitoring processed by machine learning in the neuro-ICU^30–32^.

The SHAP results inform both clinical decision-making and PCAS pathophysiology. That neurological examination and brain injury biomarkers dominate the model is consistent with current guidelines^4,6^. What is more interesting is the role of coagulation markers. Initial INR ranked 7th among 91 features—the highest-ranked variable not traditionally considered prognostic. In PCAS, coagulopathy reflects the severity of ischemia-reperfusion injury through tissue factor activation and consumptive coagulopathy^9,10^. Prior studies have confirmed the prognostic value of PT-INR^33^ and comprehensive coagulation profiles^34^ after cardiac arrest, supporting the biological plausibility of our findings. The early discriminative value of INR—universally available, inexpensive, and returned within minutes—suggests it may serve as a rapid surrogate for overall injury severity before serial biomarker data become available.

The relatively modest contribution of inflammatory markers (lowest modality-level SHAP at 0.323) was somewhat unexpected given that NLR and CRP have been linked to outcome individually^13,14^. The most likely explanation is information redundancy: coagulation and inflammation share overlapping activation pathways, so once coagulation and brain injury markers are in the model, inflammation adds little that is not already captured. This does not diminish the biological role of inflammation in PCAS; it means that for prediction, inflammatory markers are largely dispensable when richer data sources are available.

An apparent discrepancy in our results warrants explanation: SHAP analysis identifies neurological examination as a top contributor, yet excluding it reduces AUROC by only 0.018. This reflects the distinction between feature importance and feature redundancy. SHAP quantifies how much each feature contributes in the full model, where the model preferentially routes predictions through clinically intuitive variables like GCS and pupillary reflexes. When these are removed, the model compensates by upweighting brain injury biomarkers and coagulation markers, which carry overlapping information. This redundancy is precisely why the multimodal approach is robust—the loss of one data stream is buffered by correlated signals from other modalities.

The calibration analysis adds a clinically important dimension. While the random forest achieved the highest discrimination, XGBoost was better calibrated (Brier 0.050, slope 0.96). In clinical practice, where predicted probabilities inform treatment decisions, well-calibrated probabilities matter as much as raw discrimination. The overlap zone between 0.2 and 0.5 predicted probability represents patients for whom the model’s confidence is insufficient for definitive prognostication. In practice, these patients would require continued observation, serial reassessment, and integration of additional modalities (neuroimaging, continuous EEG) not currently in the model. The random forest’s tendency to compress probabilities toward the extremes would need post-hoc recalibration before any clinical deployment.

Our framework differs from EEG-based machine learning models, which have been increasingly applied to post-cardiac arrest prognostication^35^. A recent systematic review identified random forest and convolutional neural networks as the most common EEG-based approaches, with AUROCs generally in the range of 0.85–0.95. Our laboratory-and-clinical multimodal approach and EEG-based models are complementary: the former captures systemic injury severity through biomarkers and coagulation, while the latter directly assesses cortical function. Future integration of both data streams—combining serial laboratory data with continuous EEG features—could further improve prognostication.

Several methodological considerations deserve mention. We deliberately chose tree-based ensemble methods rather than deep learning architectures such as recurrent neural networks or temporal transformers^36^. With 414 patients and 91 features, deep learning models face substantial overfitting risk. That logistic regression achieved 0.972 supports the view that signal complexity is moderate. The high AUROC values (> 0.97 for all full models) reflect the strong prognostic signals inherent in this dataset: NSE and S100B trajectories, GCS, and pupillary reflexes are well-established near-deterministic predictors in post-cardiac arrest cohorts, particularly in a setting where WLST does not confound outcome ascertainment. Additionally, residual sedation effects on neurological examination cannot be entirely excluded, particularly at T0 and T24 when sedation may have been active; however, the sensitivity analysis excluding neurological examination (AUROC 0.965) shows that this potential confounding does not undermine the overall model. Our handling of missing data through median imputation may introduce bias; multiple imputation by chained equations would provide more rigorous uncertainty quantification^37^.

This study has limitations. First, the retrospective, single-center design may limit generalizability. Second, our OHCA-like benchmark is an approximation; comparisons with formally calculated scores in multicenter datasets should be pursued. Third, the 12-hour timepoint was excluded (77–94% missing), limiting early trajectory modeling^23^. Fourth, myoclonus assessment, neuroimaging (CT/MRI), and continuous EEG pattern classification were not systematically documented in our registry and therefore not integrated into the model; their inclusion could further improve performance. Fifth, although SSEP, aEEG, angiography, and PCI were classified as static variables, they reflect clinician decisions made during admission; our sensitivity analysis excluding these variables (AUROC 0.979) confirms that the model does not depend on these potentially biased features. Sixth, external validation in independent cohorts is essential. To implement such a tool clinically, it should output a continuous probability with SHAP-based explanation, serve as decision support complementing clinician judgment, and trigger continued observation for patients in the uncertain probability range (0.2–0.5). Prospective, multicenter validation with blinded prognostication is the necessary next step^38^.

Integrating serial thromboinflammation markers with brain injury biomarkers, neurological examination, and clinical variables into a single machine learning framework yields excellent discrimination for six-month neurological prognostication after OHCA (AUROC 0.983), with predictions that sharpen as data accumulate over the first 48 h and transparency through SHAP-based explainability. The Korean clinical context—where WLST is not practiced—provides a uniquely informative setting for evaluating prognostic models free from self-fulfilling prophecy. External validation in multicenter cohorts using prospective, blinded protocols is the essential next step toward clinical translation.

## Supplementary Information

Below is the link to the electronic supplementary material.

Supplementary Material 1

## Data Availability

No datasets were generated or analysed during the current study.

## Abbreviations

aEEG: Amplitude-integrated electroencephalography
ATIII: Antithrombin III
AUPRC: Area under the precision-recall curve
AUROC: Area under the receiver operating characteristic curve
CAHP: Cardiac arrest hospital prognosis
CPC: Cerebral performance category
CPR: Cardiopulmonary resuscitation
CR: Corneal reflex
CRP: C-reactive protein
EEG: Electroencephalography
FDP: Fibrin degradation products
GBM: Gradient boosting machine
GCS: Glasgow coma scale
ICU: Intensive care unit
IMRaD: Introduction methods results and discussion
INR: International normalized ratio
IQR: Interquartile range
LR: Logistic regression
NLR: Neutrophil-to-lymphocyte ratio
NSE: Neuron-specific enolase
OHCA: Out-of-hospital cardiac arrest
PCAS: Post-cardiac arrest syndrome
PCI: Percutaneous coronary intervention
PLR: Pupillary light reflex
PT: Prothrombin time
RF: Random forest
ROSC: Return of spontaneous circulation
S100B: S100 calcium-binding protein B
SD: Standard deviation
SHAP: SHapley Additive exPlanations
SSEP: Somatosensory evoked potentials
STEMI: ST-elevation myocardial infarction
TRIPOD: Transparent reporting of a multivariable prediction model for individual prognosis or diagnosis
TTM: Targeted temperature management
WLST: Withdrawal of life-sustaining treatment
XGBoost: Extreme gradient boosting

## Code availability

The underlying code for this study is not publicly available but may be made available to qualified researchers on reasonable request from the corresponding author.
